# Analyzing the changing relationship between personal consumption and suicide mortality during COVID-19 pandemic in Japan, using governmental and personal consumption transaction databases

**DOI:** 10.3389/fpubh.2022.982341

**Published:** 2022-09-07

**Authors:** Ryusuke Matsumoto, Yasuhiro Kawano, Eishi Motomura, Takashi Shiroyama, Motohiro Okada

**Affiliations:** Department of Neuropsychiatry, Division of Neuroscience, Graduate School of Medicine, Mie University, Tsu, Japan

**Keywords:** COVID-19, Japan, lifestyle, personal consumption, suicide

## Abstract

During the early stages of the ongoing COVID-19 pandemic, suicides did not increase in most countries/regions. Japan, however, was an exception to this, reporting increased numbers of female suicides with no changes in male suicide. To explore the trends of increasing suicides, the fluctuations of personal consumption (as an indicator of lifestyle) and standardized suicide death rate (SDR) disaggregated by age, sex, and prefecture, were determined using a linear mixed-effect model. Additionally, fixed effects of personal consumption on SDR during the pandemic were also analyzed using hierarchical linear regression models with robust standard errors. During the first wave of the pandemic, SDR for both sexes decreased slightly but increased during the second half of 2020. SDR of females younger than 70 years old and males younger than 40 years old continued to increase throughout 2021, whereas SDR for other ages of both sexes did not increase. Personal consumption expenditures on out-of-home recreations (travel agencies, pubs, and hotels) and internet/mobile communication expenses decreased, but expenditures on home-based recreations (contents distribution) increased during the pandemic. Increased expenditures on internet/mobile communication were related to increasing SDR of both sexes. Increasing expenditures on content distributions were related to increasing females' SDR without affecting that of males. Decreasing expenditures on pubs were related to increasing SDR of both sexes in the non-metropolitan region. These findings suggest that transformed individual lifestyles, extended time at home with a decreased outing for contact with others, contributed to the progression of isolation as a risk of suicide. Unexpectedly, increasing compensatory contact with others using internet/mobile communication enhanced isolation resulting in increased suicide risk.

## Introduction

In the early months of the coronavirus disease 2019 (COVID-19) pandemic, psychiatrists and psychologists expressed their concern that the ongoing pandemic would adversely affect both socio-psychological and socio-economic status in unprecedented ways (1–4). Various studies have revealed that the pandemic has negatively affected the economy and education, in addition to public health and medical care (5, 6). Likewise, the mental health of people globally has deteriorated due to increased levels of anxiety regarding the disease itself, as well as lifestyle changes caused by government-imposed social restrictions. Such restrictions have led to increasing unemployment and uncertainties about access to medical care (1). It is easily speculated that such deterioration in the psychosocial conditions of individuals would be correlated to increases in suicide mortality (1). However, unexpectedly, such increases in rates of suicide mortality were not observed in most upper-middle income countries/regions in 2020, with the exception of Japan and Vienna, Austria (7–15). Some countries even experienced decreases in the rates of suicide following the start of the pandemic. In the United States, for example, suicide mortality rates had been increasing in previous years, but actually decreased in 2020 in comparison to 2019 (16). Japan and India, on the other hand, had previously experienced decreasing trends in suicide mortality prior to the pandemic but saw an increase in suicide mortality from 2019 to 2020 (15, 17).

Several reports suggested that the governmental restriction measures to prevent the spread of COVID-19 in the communities possibly suppressed not only COVID-19 but also suicide *via* the countermeasures against restriction measures, such as support for economic and mental health, in Germany, Canada, and the United States, where a decrease was seen in the number of suicides during the pandemic (7, 18–20). However, the biased targeting of relief efforts was emphasized by the relative increasing suicide mortality in the racial minority (19, 20). The Japanese government had also responded to the socio-economic/socio-psychological deteriorations induced by the COVID-19 pandemic itself and/or social restriction measures (21). Indeed, the Ministry of Health, Labor and Welfare (MHLW) quickly budgeted for “Suicide prevention measures in response to the COVID-19 pandemic,” in both 2020 and 2021, since MHLW speculated the increasing suicides due to the socio-psychological/socioeconomic deteriorations induced by the COVID-19 pandemic (21). “Suicide prevention measures in response to the COVID-19 pandemic” was an essentially enhanced program of the conventional governmental “Regional comprehensive suicide prevention program,” since its major activities are carried by the public service corporations, social welfare corporations, and NPOs that have contributed to “Emergency Fund to Enhance Community-Based Suicide Countermeasures (21).” These findings in the Western countries and Japan suggest the importance of targets for relief efforts as part of countermeasures against socio-economic and socio-psychological deteriorations during the pandemic. In other words, some novel socio-psychological mechanisms for increasing suicides during the pandemic, which were not covered by the conventional suicide prevention programs might have been generated during the pandemic in Japan (14, 15), if any, they are possibly heterogeneous forms of national, ethnic, or personal cultural factors different from the traditional risk factors for suicide (22–25).

Several studies have tried to identify the features responsible for the increased suicides during the pandemic phase in Japan (12, 14, 15, 26, 27) because this increase was associated with various complicated factors (12, 14, 26, 28, 29). Until recently, various risk factors for an increase in suicide numbers during the pandemic were reported (12, 14, 15, 26, 27). First, Werther's effect (copycat suicide) (30) was seen to have a significant role in the transient/drastic increase in suicides in October 2020 (12, 15). It has been speculated that the spread of information on internet communications induced by mass media has probably been involved in Werther's effect in October 2020 (12, 15, 31). Second, during 2009–2019, decreased suicides among the elderly contributed to a decrease in the national-level suicide rates (24, 25, 32, 33), whereas during the pandemic, suicides among working-age individuals notoriously increased (12, 14, 15, 26). An increase in suicides among younger age groups (aged 20–29) during the pandemic is peculiar in light compared to pre-pandemic (14, 27). Third, although the specific motives for increasing suicides could not be detected (12, 14), suicides at their living place (at home) and by hanging [traditionally the most frequent suicide method in Japan (23, 32)] among females during the pandemic accounted the for the increasing suicide numbers in Japan (14, 15). Although these factors for suicide during the pandemic are fragmented, several specific features, such as an increase in suicides at home and by hanging (frequently carried out at their home) and the contribution of information diffusion *via* the Internet (predominantly exposure to younger population) indicate the possibility that specific transformed lifestyle changes, such as extended time at home (or decreased outing for contact with others) and decrease in face-to-face contact (or increasing contact *via* the Internet), resulted in increased suicide risk *via* socio-psychological deteriorations of individuals. The intensity of restriction measures predominantly deteriorated the mental health of individuals rather than the duration of restriction (repeated/intermittent restriction measures deteriorated the mental health of the same individuals) (4, 34, 35). However, taken together with the fact that fewer numbers of patients were infected with and died from COVID-19 in Japan, along with milder social restriction politics compared to those in Europe (36–38), these findings also suggest that the transformation of lifestyle plays a salient role in increasing the suicides during the pandemic.

Several studies have revealed that individuals experienced changes in their daily lifestyles during the pandemic; for instance, an increase in online activities replaced physical participation and contributed to changes in urban mobility (39–42). During the restriction on restaurants/pubs (April-June 2020), the night-time population in Tokyo decreased (40). Online communications also contributed to the maintenance of business, education, consumption, and medicine. Additionally, behavioral changes due to governmental social restriction measures were also reported (39). It is well known that consumer behavior is influenced by various factors, including purchasing power/capacity, purchasing intentions, preference, and daily necessities (41–48). Therefore, changes in personal consumption behaviors can reflect the consequences of social, economic, and psychological factors that have transformed during the pandemic. It can be speculated that the pandemic has probably affected personal consumption behavior, since consumption is a prosperity indicator of a variety of lifestyles, from food and daily necessities to recreation (46–48). However, there are no reports of quantitative analysis of changing lifestyles and/or personal consumption in Japan during the pandemic. Real-time monitoring of suicide mortality represents a critical goal for public health efforts. Considering these psycho-social/socio-economic findings, we hypothesized that transformed lifestyles during the pandemic probably played an important role in the increased suicide pandemic in Japan. Therefore, to clarify the relations between transformed lifestyles and suicide mortality, the present study determined the temporal fluctuations of monthly personal consumption expenditures (as lifestyle indicators) disaggregated by prefecture, and monthly age-standardized death rate of suicide (SDR) disaggregated by sex, age, and prefecture using a linear mixed-effect model. Furthermore, the relationship between personal consumption and SDR was determined using fixed-effects of hierarchical linear regression models with robust standard errors.

## Methods

### Data sources

The monthly reports of suicide in each prefecture were disaggregated by sex (i.e., male and female) and age (i.e., younger than 20 years old [10s], 20–29 [20s], 30–39 [30s], 40–49 [40s], 50–59 [50s], 60–69 [60s], 70–79 [70s], and over 80 years old [80s]). This data was obtained from the Basic Data on Suicide in the Region (BDSR) database, generated by the Japan Ministry of Health, Labor, and Welfare (MHLW) (49). The prefectural populations disaggregated by sex and age were obtained from the Regional Statistics Database (RSD) of the System of Social and Demographic Statistics generated by the Statistics Bureau of the Ministry of Internal Affairs and Communications (SBMIAC) (50).

The personal consumption data were obtained from the “JCB Consumption NOW” database (Nowcast, Tokyo, Japan) (51). The governmental consumption statistics disaggregated by prefectures were obtained from the “Family Income and Expenditure Survey (24, 52)” and the “Current Survey of Commerce (53)” published by SBMIAC. The “Family Income and Expenditure Survey” and the “Current Survey of Commerce” involved a questionnaire survey of 9,000 households and 15,000 shops, respectively (24, 52, 53). JCB Consumption NOW provides the credit card transaction data of JCB (JCB Co Ltd, Tokyo, Japan) (note, the number of card members and annual transaction volume of JCB globally in 2020 were 141 million people and 305.5 billion USD, respectively) (54). In contrast to governmental consumption statistics, consumption statistics from JCB Consumption NOW are obtained from the actual purchase (with the exception of purchases made by members that did not authorize the use of their data). During such a purchase, purchase information, such as how much a consumer spent at which store on which day, is recorded (51). The consumption amount of the consumers subject to surveys of “Family Income and Expenditure Survey” and “JCB Consumption NOW” are 30 million JPN (9 thousand households) and 4 trillion JPN (more than 10 million persons), respectively (51, 52). Importantly, any information in JCB Consumption NOW is provided on the premise that measures are taken to maintain confidentiality by making it impossible to identify individuals so that the day-to-day purchasing behavior of individuals cannot be traced. Indeed, due to this usefulness/effectiveness compared to governmental consumption statistics, JCB Consumption NOW has been adopted by the RESAS database “Regional Economy and Society Analyzing System” and V-RESAS database “Vital Signs of Economy Regional Economy and Society Analyzing System” published by Cabinet Office (55, 56). In the present study, individual consumption refers to expenditures by credit card for tangibles, cars, retails (cloth, food/drink, clothes, supplement, non-prescription drugs, cosmetics,), services (pub, travel agency, hotel), medical expenditures (medical facilities), stores (supermarkets, shopping malls, and convenience stores), communication expense, contents distribution and lifelines (electric, waters, fuel charges), and total personal expenditures. All monthly individual consumption data between January 2017 and December 2021 were provided as relative values disaggregated by prefectures against March 2016 data.

Although the need for ethical approval and informed consent was exempted by the Medical Ethics Review Committee of Mie University due to the use of publicly available data, this study adhered to the Strengthening the Reporting of Observational Studies in Epidemiology (STROBE) guidelines. There were no missing independent or dependent values in this study.

### Monthly age-standardized death rate of suicide

Prefectural crude suicide rates disaggregated by sex and age were calculated by dividing the monthly numbers by prefecture population in the same year. These values were calculated using the empirical Bayes standardized mobile ratio method with an empirical Bayes estimator for the Poisson/gamma model (ver 2.1; National Institute of Public Health, Wako, Japan; https://www.niph.go.jp/soshiki/gijutsu/download/ebpoig/index_j.html; accessed 1 January 2022) to eliminate artifacts induced by small prefectural populations. The age-standardized death rate by suicide (SDR) for males and females per 100,000 of the population was calculated based on the 2019 Japanese age-dependent population composition since the age distributions between the WHO standard population model and Japan differ (33, 57). According to previous studies, all 47 prefectures were categorized as metropolitan regions, including the Capital (Kanto) region (Tokyo-to, Saitama-ken, Chiba-ken, and Kanagawa-ken), Kansai region (Osaka-fu, Kyoto-fu, and Hyogo-ken), Tokai region (Aichi-ken), Fukuoka area (Fukuoka-ken) and Sapporo area (Hokkai-do) and non-metropolitan region (14, 33, 57).

### Survey of periods for deriving the predicted values

Suicide statistical analysis during the COVID-19 pandemic should adopt the value pre-pandemic and/or predicted value using time series models as control data since there are no actual control data. When comparing the suicide rate relative to previous years in the same month, the results may be confounded by a long-term ascending or descending trend (13–15). Indeed, suicide mortality in Japan has continued to decrease during 2009–2019, therefore, the possibility of overestimating the decrease and underestimating the increase during the pandemic cannot be ignored when targeting pre-pandemic data (15). Recently, the interrupted time series analysis (ITSA) has been considered to be the most reliable method for analyzing suicide data during the COVID-19 pandemic (1). Both the reports using ITSA detected excess suicide rates during the second half of 2020 in Japan; however, one report (study period: between November/2016 and October/2020) (13) detected a lower suicide rate during the first wave of the pandemic (March-June/2020), whereas another report (study period: between January/2010 and May/2021) (12) could not find such changes. This discrepancy suggests the importance of appropriate survey periods in study design, based on pre- and post-comparison. Indeed, the average suicide rate in 2019 was 6.4% and 25%, lower compared to 2017 and 2013, respectively (13). Furthermore, during 2017–2019, the fluctuation of suicides in Japan displayed a seasonally increase in March and then a slight decrease (13–15). Therefore, to derive the actual predicted value, a method that fully reflects seasonal fluctuation factors should be used to derive the predicted value (13–15). Based on these methodological backgrounds, to identify the appropriate period for calculating predicted SDR during the pandemic, the joinpoint of SDR during 2009–2019 was analyzed by ITSA (Joinpoint Regression Program v4.9.0.0, National Cancer Institute, Bethesda MD) (15). According to the joinpoint period of ITSA analysis, the predicted SDRs and expenditures of personal consumption between January 2020 and December 2021 were calculated by the seasonal autoregressive integrated moving average method (observation data = trend variation ^*^ cyclical variation ^*^ seasonal variation ^*^ irregular variation) (Bell Curve for Excel v.3.22, Social Survey Research Information Co., Tokyo, Japan; RRID: SCR_017294) (15, 58).

### Statistical analysis

Prefectural monthly SDR and personal consumption expenditures between the predicted and observed values between 2020 and 2021 were compared using a linear mixed-effect model by SPSS for Windows version 27 (IBM, Armonk, NY, USA) (15, 59). When the data did not violate the assumption of sphericity (*p* > 0.05), F-value of the linear mixed-effect model was analyzed using sphericity-assumed degrees of freedom, whereas if the assumption of sphericity was violated (*p* < 0.05), *F*-value was analyzed using Greenhouse-Geisser's corrected degrees of freedom. When F-value was significant (*p* < 0.05), data were analyzed using Scheffe's *post-hoc* analysis (15, 59).

The impact of the personal consumption on SDR during the pandemic (between March/2020 and December/2021) was determined by the fixed-effect of a hierarchical linear regression model with robust standard error using gretl v2021d (accessed on 28 December/2021) (15). Initially, to prevent multicollinearity, the present study analyzed the variance inflation factor, and values of any factors with a variance inflation factor < 10 were adopted for analyses (33, 57). Following panel data applications (hierarchical linear regression model), the regression model was: SDR = γ_00+_ $\overset{n}{\underset{i=1}{\sum }}$($\gamma_{10^{*}n}^{*}$(expenditures)ij + $\gamma_{10^{*}n+1}$ ^*^ (centered_expenditures)_j_^*^ (expenditures)ij + u_0j_ + r_ij_ (residual), where (expenditures)_i_ was the value of personal consumption expenditures, such as credit card, tangibles, retail, cars, service (total), supermarket, shopping mall, convenience store, clothes, food/drink, lifelines, supplement, non-prescription drug, medical, pub, travel agency, hotel, cosmetic, communication and contents distribution. Although the fixed-effects model can control for unobserved time-invariant factors, such as culture, climate, economic and educational situation, etc., (also including sleep, diet, exercise, and social rhythms), that may affect the incidence or mortality induced by COVID-19, resulting in an effect on regional suicide mortality rates each month, the present study adopted robust standard errors clustered by prefectures to prevent heteroscedasticity and autocorrelation (14, 15, 22, 23). Fixed-effects were applied to the hierarchical linear regression analysis when Hausman's likelihood ratio test indicated statistical significance (*p* < 0.05) (15).

## Results

### Survey of periods for deriving the predicted values

ITSA detected the joinpoint of SDR on 2017 (slopes:−5.53 and−2.42 during 2009–2017 and 2017–2019, respectively) (Supplementary Figure S1A). According to these results, predicted SDRs between January 2020 and December/2021 were calculated from observed SDRs between January 2017 and December 2019 (Supplementary Figure S1B).

### Suicide mortality

**Figure 1:**
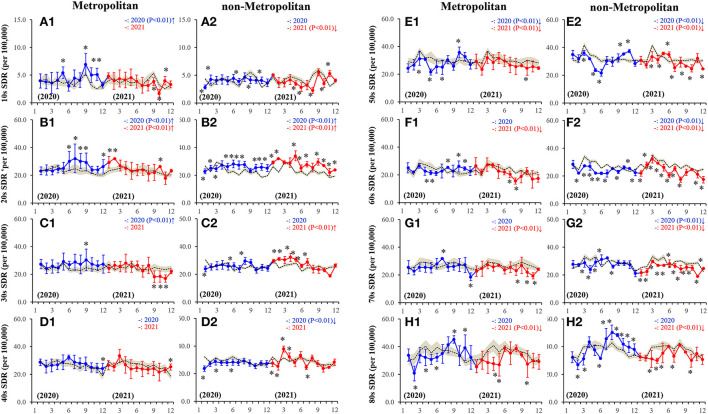
Fluctuations of monthly males SDR disaggregated by regions, metropolitan **(A1-H1)** and non-metropolitan **(A2-H2)** regions, and ages, 10s **(A1-A2)**, 20s **(B1-B2)**, 30s **(C1-C2)**, 40s **(D1-D2)**, 50s **(E1-E2)**, 60s **(F1-F2)**, 70s **(G1-G2)** and 80s **(H1-H2)**, during the COVID-19 pandemic (2020–2021). Dotted, blue and red lines indicate the average predicted and observed SDRs in 2020 and 2021, respectively. Brown areas, blue and red bars indicate the 95% CI for the predicted and observed SDRs in 2020 and 2021, respectively. Ordinates indicate the standardized suicide mortality (SDR per 100,000 people), and abscissas indicate the month. *p < 0.05, significant change of monthly SDR in comparison to predicted SDR using a linear mixed-effects model with Scheffe's *post-hoc* test. ↑and↓indicate significantly higher and lower observed annual SDR in comparison to predicted annual SDR, respectively.

During the first wave pandemic (between March-June 2020), the monthly SDRs for males aged the 50s−60s and 80s in the metropolitan region and of those aged 40s−80s in the non-metropolitan region were lower than predicted values, but higher SDRs were not observed in all ages (Figure 1). During the second half of 2020, in the metropolitan region, the higher SDRs were detected in some months compared to the predicted SDR of all ages (Figure 1). In the non-metropolitan region, except for those in their 40s or 70s, higher SDRs were detected in some months compared to the predicted SDR, whereas the lower SDRs of those aged 10s and those aged 60s−70s were observed in some months (Figure 1). In the first half of 2021, the higher SDRs of those in their 20s in the metropolitan region and of those aged 20s−40s in the non-metropolitan region were observed. However, the SDRs of those aged 10s and 50s−80s in non-metropolitan regions were lower than predicted SDRs. Contrarily, in the second half of 2021, except for those in the 20s, the SDRs in both regions were lower than the predicted SDR in some months (Figure 1).

**Figure 2:**
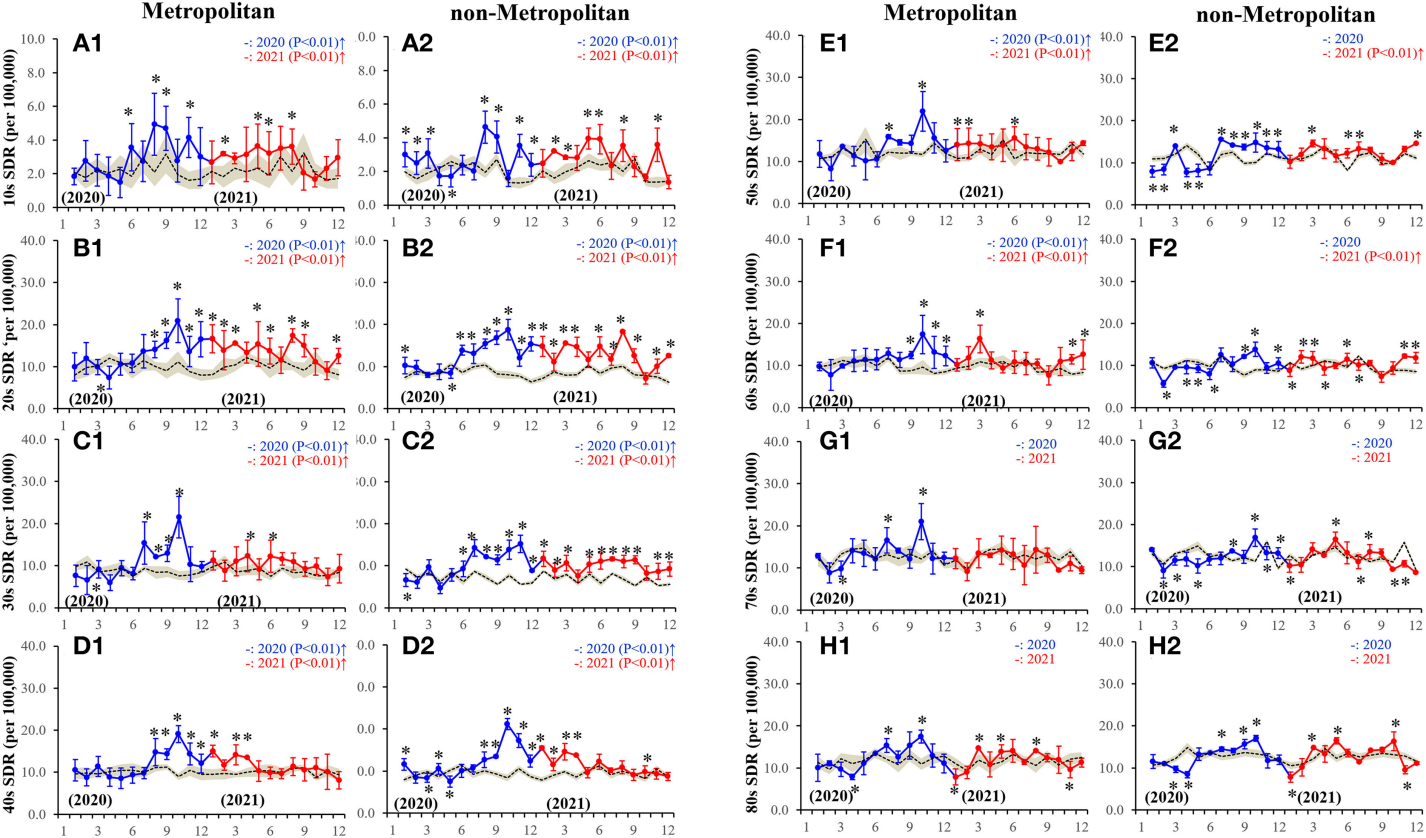
Fluctuations of monthly females SDR disaggregated by regions, metropolitan **(A1-H1)** and non-metropolitan **(A2-H2)** regions, and ages, 10s **(A1-A2)**, 20s **(B1-B2)**, 30s **(C1-C2)**, 40s **(D1-D2)**, 50s **(E1-E2)**, 60s **(F1-F2)**, 70s **(G1-G2)** and 80s **(H1-H2)**, during the COVID-19 pandemic (2020–2021). Dotted, blue and red lines indicate the average predicted and observed SDRs in 2020 and 2021, respectively. Brown areas, blue and red bars indicate the 95% CI for predicted and observed SDR in 2020 and 2021, respectively. Ordinates indicate the standardized suicide mortality (per 100,000 people), and abscissas indicate the month. *p < 0.05, significant change of monthly SDR in comparison to predicted SDR using a linear mixed-effects model with Scheffe's *post-hoc* test. ↑and↓indicate significantly higher and lower observed annual SDR in comparison to predicted annual SDR, respectively.

From March-June 2020, the monthly SDRs for females were observed to not be higher (lower or nearly equal) than the predicted SDR. During the second half of 2020, the SDRs of all age groups in both metropolitan and non-metropolitan regions were higher than the predicted SDRs (Figure 2). The peaks in SDRs of almost all ages were observed in October/2020 (Figure 2). In the first half of 2021, the higher SDRs of individuals younger than 60 in both regions were observed (Figure 2). However, the SDRs of those aged in their 10s−20s in the metropolitan region and between 10s and 30s in the non-metropolitan region continued to be higher than predicted SDR (Figure 2).

In 2020, in the metropolitan region, the annual male SDRs of those aged 10s−30s and those in their 50s were higher and lower than predicted SDRs, respectively; however, in the non-metropolitan region, the annual SDRs of those in their 20s and those aged 40s−70s were higher and lower than predicted SDRs, respectively (Supplementary Figure S2). In 2021, in the metropolitan region, the annual SDRs of the 20s alone and 50s−80s were higher and lower than the predicted SDR, respectively (Supplementary Figure S2). In the non-metropolitan region, the annual SDRs of those aged in their 20s−30s were higher, but of those in the age range between 10s and 50s−80s were lower than predicted (Supplementary Figure S2). The annual SDRs of those aged in their 10s in both the regions in 2021 were lower than that in 2020 (Supplementary Figure S2). Conversely, in the non-metropolitan region, the SDRs of those aged the 20s−40s in 2021 were higher, but of those aged above 50 years in 2021 were lower than in 2020 (Supplementary Figure S2). Annual SDRs for males aged from 10s to 80s in 2021 were lower than in 2020 in both regions (Supplementary Figure S2). In non-metropolitan regions, annual SDRs for males aged the 20s−40s and 50s−70s in 2021 were higher and lower than in 2020, respectively (Supplementary Figure S2).

In the metropolitan region, the annual SDRs for females aged 10s−60s in both 2020 and 2021 were higher, but of those aged 70s−80s were nearly equal to the predicted SDR (Supplementary Figure S2). In the non-metropolitan region, annual SDRs for females aged between 10s−40s in 2020 and 2021 were higher than predicted SDRs, but of those aged 50s−60s in 2021 alone were higher than predicted SDRs (Supplementary Figure S2). Especially, in the non-metropolitan region, the annual SDRs of those in their 20s and 50s in 2021 were higher than in 2020 (Supplementary Figure S2). During 2020–2021, annual females SDRs of the 20s−30s in the non-metropolitan region were higher than those in the metropolitan region (Supplementary Figure S2). In the non-metropolitan region, annual female SDRs in 20s and 50s in 2021 were higher than those in 2020 (Supplementary Figure S2).

### Personal consumption expenditures

**Figure 3:**
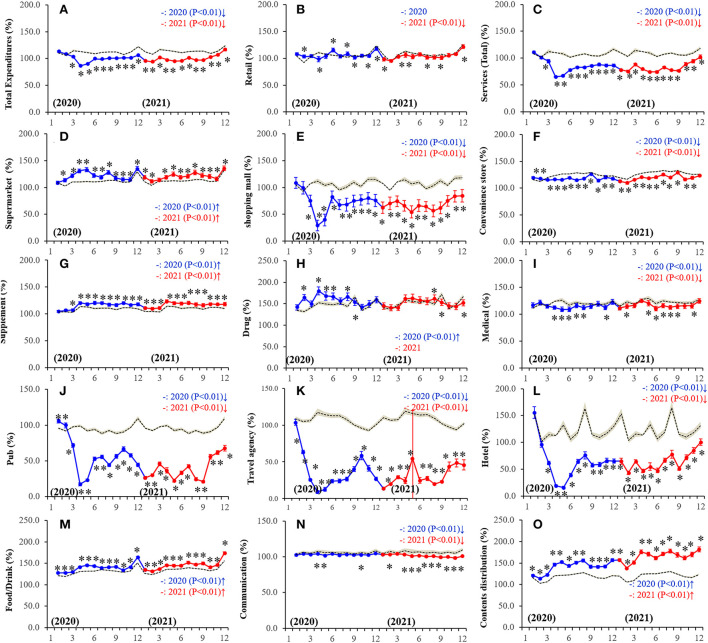
Fluctuations of monthly expenditures during the COVID-19 pandemic in Japan. **(A)** total expenditure, **(B)** retail, **(C)** service (total), **(D)** supermarket, **(E)** shopping mall, **(F)** convenience store, **(G)** supplement, **(H)** drug (nonprescription drug), **(I)** medical, **(J)** pub, **(K)** travel agency, **(L)** hotel, **(M)** food/drink, **(N)** communication, and **(O)** contents distribution. Ordinates indicate the relative personal expenditures between January 2020 and December 2021 per that in March 2016 (%), and abscissas indicate the month. Dotted, blue, and red lines indicate the average predicted and observed personal expenditures in 2020 and 2021, respectively. Brown areas, blue and red bars indicate the 95% CI for predicted and observed personal expenditures in 2020 and 2021, respectively. *p < 0.05, significant change of monthly personal consumption expenditures in comparison to predicted vales using a linear mixed-effects model with Scheffe's *post-hoc* test. ↑and↓indicate significantly higher and lower observed monthly expenditures in comparison to predicted monthly expenditures, respectively.

During January-February 2020 (period of the pandemic onset), total personal expenditure was almost equal but became lower than the predicted values during the pandemic (Figure 3). From January-February 2020, personal expenditure to service (total) was also almost equal but became lower than the predicted values during the pandemic (Figure 3). Most notable changes were expenditures toward out-of-home recreations, such as travel agencies, hotels, and pubs, which decreased drastically around the initiation of the pandemic and reduction continued throughout the pandemic, whereas expenditures to content distributions (home-based recreation) increased compared to predicted values (Figure 3; Supplementary Figure S4). Furthermore, a transient increase in consumption in pubs and travel agency were observed in October 2020 (Figure 3), when female SDR increased transiently/drastically (Figure 2). Increased home-based and decreased out-of-home recreational expenditures were more pronounced in 2021 than in 2020 (Figure 3; Supplementary Figure S4).

Expenditures of communication expenses decreased during the pandemic in metropolitan regions, whereas in the non-metropolitan region, communication expenses slightly increased and then decreased in 2020 and 2021, respectively, but the amount was observed to be almost equal to the predicted value (Figure 3; Supplementary Figure S4). Retail consumption was nearly equal in 2020 but slightly lower in 2021 than predicted values (Figure 3; Supplementary Figure S4). Consumptions in shopping malls and convenience stores were lower, but conversely, consumption in supermarkets was higher than predicted during the pandemic (Figure 3; Supplementary Figure S4). Expenditures for daily necessary food/drink continuously increased during the pandemic, similar to consumption in supermarkets (Figure 3; Supplementary Figure S4). Consumptions in shopping malls and convenience stores were lower, but conversely, consumption in supermarkets was higher than predicted during the pandemic (Figure 3; Supplementary Figure S4). Expenditures on both supplements and non-prescription drugs that could be bought online were higher, but medical expenditures, for which individuals must visit medical facilities, were lower than predicted (Figure 3; Supplementary Figure S4).

### Fixed-effects of personal consumption expenditures on SDR during the pandemic

Fixed-effect of a hierarchical linear regression model with a robust standard error detected the significant relations between several personal consumption expenditures and SDR. Males SDR in the metropolitan region was positively related to expenditures on communication expenses but was negatively related to expenditure on the credit card (Table 1). Females SDR in the metropolitan region was positively related to expenditures on retail, cars, supermarket, food/drink, travel agencies, communication, and contents distribution, but was negatively related to expenditure on tangibles and lifelines (Table 1). Males SDR in the non-metropolitan region was positively related to expenditures on retail and contents distribution but was negatively related to expenditure on supermarkets, shopping malls, food/drink, and pub (Table 2). Females' SDR in the non-metropolitan region was positively related to expenditures on cars, cloth, food/drink, communication, and contents distribution, but was negatively related to expenditure on tangibles, supermarket, lifelines, and pubs (Table 2). Considering the fluctuation of personal expenditures, the increased expenditure on content distribution during the pandemic (Figure 3) contributed to increasing female SDR but did not affect male SDR in both metropolitan and non-metropolitan regions (Tables 1, 2). On the contrary, drastically decreasing expenditure on pubs during the pandemic (Figure 3) did not affect SDRs of males and females in metropolitan regions, but contributed to increasing SDRs of both males and females in the non-metropolitan region (Tables 1, 2). The positive impacts of expenditure to travel agencies on female SDR in the metropolitan region was neutralized by the drastically decreasing expenditure during the pandemic (Figure 3).

**Table 1:** Relationship between personal consumption expenditures and SDRs for males and females, only males, and only females in the metropolitan region during the COVID-19 pandemic (between March 2020 and December 2021).

**Factor**	**Males** + **Females**				**Males**				**Females**			
	**β**	**SE**	**T**	***p***	**β**	**SE**	**T**	***p***	**β**	**SE**	**T**	***p***
Credit card	−0.123	0.049	−2.531	0.012*	−0.204	0.083	−2.454	0.037*	−0.046	0.030	−1.554	0.122
Tangibles	−0.237	0.236	−1.004	0.316	0.078	0.348	0.225	0.827	−0.537	0.197	−2.725	0.007**
Retail	0.143	0.063	2.272	0.024*	0.110	0.082	1.342	0.213	0.174	0.050	3.452	0.001**
Cars	0.028	0.033	0.863	0.389	−0.021	0.044	−0.474	0.647	0.075	0.027	2.818	0.005**
Service (total)	−0.022	0.035	−0.623	0.534	0.000	0.081	0.000	1.000	−0.043	0.027	−1.571	0.118
Supermarket	−0.016	0.032	−0.508	0.612	−0.090	0.043	−2.104	0.065	0.054	0.026	2.077	0.039*
Shopping mall	0.006	0.027	0.227	0.821	−0.045	0.030	−1.505	0.167	0.055	0.031	1.759	0.080
Convenience store	−0.056	0.045	−1.250	0.213	−0.020	0.047	−0.426	0.680	−0.090	0.051	−1.779	0.077
Clothes	0.062	0.033	1.865	0.064	0.086	0.049	1.747	0.115	0.039	0.042	0.943	0.347
Food/drink	0.099	0.095	1.037	0.301	0.021	0.134	0.155	0.880	0.173	0.077	2.258	0.025*
Lifelines	−0.035	0.009	−3.903	0.000**	−0.032	0.014	−2.212	0.054	−0.038	0.013	−2.996	0.003**
Supplement	0.030	0.043	0.688	0.492	0.003	0.059	0.053	0.959	0.055	0.041	1.364	0.174
Non-prescription drug	−0.013	0.024	−0.528	0.598	−0.034	0.028	−1.245	0.244	0.008	0.022	0.374	0.709
Medical	0.023	0.033	0.693	0.489	0.033	0.059	0.562	0.588	0.013	0.026	0.502	0.616
Pub	−0.008	0.016	−0.491	0.624	0.014	0.022	0.643	0.536	−0.029	0.019	−1.549	0.123
Travel agency	0.026	0.013	1.942	0.054	−0.001	0.027	−0.028	0.978	0.052	0.012	4.475	0.000**
Hotel	0.004	0.009	0.403	0.687	0.008	0.017	0.474	0.647	0.000	0.012	−0.022	0.982
Cosmetic	−0.036	0.016	−2.267	0.024*	−0.038	0.025	−1.515	0.164	−0.034	0.022	−1.557	0.121
Communication	0.320	0.077	4.137	0.000**	0.325	0.096	3.378	0.008**	0.316	0.100	3.172	0.002**
Contents distribution	0.049	0.019	2.654	0.009**	0.037	0.035	1.068	0.314	0.061	0.021	2.885	0.004**
Hausman X^2^=	119.89			*(p < 0*.01)	112.37			*(p < 0*.01)	152.31			*(p < 0*.01)

β, coefficient value; SE, standard error; T, T vales; X^2^, Chi-square value by Hausman's likelihood ratio test.

* p < 0.05,

** p < 0.01.

**Table 2:** Relationship between personal consumption expenditures and SDRs for males and females, only males, and only females in the non-metropolitan region during the COVID-19 pandemic (between March 2020 and December 2021).

**Factor**	**Males** + **Females**				**Males**				**Females**			
	**β**	**SE**	**T**	***p***	**β**	**SE**	**T**	***p***	**β**	**SE**	**T**	***p***
Credit card	0.030	0.022	1.379	0.176	0.024	0.028	0.849	0.402	0.037	0.024	1.509	0.140
Tangibles	−0.033	0.048	−0.680	0.501	0.098	0.084	1.173	0.249	−0.157	0.046	−3.409	0.002**
Retail	0.037	0.018	2.033	0.049*	0.049	0.024	2.057	0.047*	0.026	0.018	1.442	0.158
Cars	0.009	0.007	1.260	0.216	−0.010	0.011	−0.849	0.401	0.026	0.007	3.875	0.000**
Service (total)	0.006	0.006	1.098	0.280	0.005	0.010	0.479	0.635	0.008	0.009	0.939	0.354
Supermarket	−0.056	0.017	−3.343	0.002**	−0.084	0.024	−3.571	0.001**	−0.029	0.014	−2.069	0.046*
Shopping mall	−0.023	0.005	−4.243	0.000**	−0.037	0.009	−4.194	0.000**	−0.010	0.006	−1.552	0.129
Convenience store	0.016	0.012	1.316	0.196	0.035	0.018	1.944	0.060	−0.002	0.014	−0.157	0.876
Clothes	0.001	0.010	0.141	0.889	−0.026	0.017	−1.601	0.118	0.028	0.010	2.734	0.010*
Food/drink	−0.024	0.020	−1.216	0.232	−0.094	0.035	−2.646	0.012*	0.042	0.017	2.451	0.019*
Lifelines	−0.018	0.003	−6.651	0.000**	−0.001	0.004	−0.324	0.748	−0.034	0.004	−9.452	0.000**
Supplement	0.002	0.007	0.338	0.737	−0.006	0.010	−0.568	0.574	0.010	0.009	1.127	0.267
Non-prescription drug	−0.012	0.007	−1.844	0.073	−0.007	0.006	−1.081	0.287	−0.017	0.009	−1.996	0.054
Medical	0.004	0.003	1.057	0.297	0.009	0.005	1.725	0.093	−0.001	0.004	−0.220	0.827
Pub	−0.017	0.005	−3.250	0.003**	−0.018	0.008	−2.301	0.027*	−0.017	0.005	−3.460	0.001**
Travel agency	0.000	0.000	−1.379	0.176	−0.001	0.000	−1.468	0.151	0.000	0.000	−0.458	0.650
Hotel	0.002	0.003	0.853	0.400	−0.002	0.004	−0.652	0.518	0.007	0.004	1.780	0.084
Cosmetic	−0.002	0.002	−1.131	0.266	−0.002	0.003	−0.553	0.584	−0.003	0.003	−1.114	0.273
Communication	0.134	0.024	5.619	0.000**	0.169	0.030	5.543	0.000**	0.100	0.027	3.720	0.001**
Contents distribution	0.003	0.005	0.727	0.472	−0.008	0.008	−1.079	0.288	0.014	0.005	2.824	0.008**
Hausman X^2^=	214.61			*(p < 0*.01)	246.39			*(p < 0*.01)	152.83			*(p < * 0.01)

β, coefficient value; SE, standard error; T, T vales; X^2^, Chi-square value by Hausman's likelihood ratio test.

* p < 0.05,

** p < 0.01.

Fixed-effect of a hierarchical linear regression model with robust standard error also detected the significant relations between several personal consumption expenditures and the SDR of individuals in their 20s. SDR of 20s females in the metropolitan region was positively related to expenditures on cars, shopping malls, food/drink, non-prescription drugs, communication, and contents distribution, but was negatively related to expenditure on tangibles, whereas 20s males' SDR did not relate to any personal consumption expenditures (Table 3). Conversely, 20s male SDR in the non-metropolitan region was positively related to expenditures on lifelines and medical but was negatively related to expenditure on supermarkets, food/drink, and pubs (Table 4). Females' SDR in the non-metropolitan region was positively related to expenditures on clothes, communication, and content distribution, but was negatively related to expenditures on credit cards and pubs (Table 4). Considering the fluctuation of personal expenditures, the increased expenditures on content distribution during the pandemic (Figure 3) contributed to increasing 20s female SDR in both metropolitan and non-metropolitan regions but did not affect 20s males SDR in both regions (Tables 3, 4). On the contrary, drastically decreasing expenditures on pubs during the pandemic (Figure 3) did not affect the 20s SDRs of males and females in metropolitan regions, but contributed to increasing 20s SDRs of both males and females in the non-metropolitan region (Tables 3, 4).

**Table 3:** Relationship between personal consumption expenditures and SDRs for males and females in their twenties in the metropolitan region during the COVID-19 pandemic (between March 2020 and December 2021).

**Factor**	**Males**				**Females**			
	**β**	**SE**	**T**	***p***	**β**	**SE**	**T**	***p***
Credit card	−0.239	0.113	−2.115	0.064	−0.027	0.096	−0.281	0.785
Tangibles	0.243	0.984	0.247	0.811	−1.944	0.404	−4.817	0.001**
Retail	0.066	0.202	0.326	0.752	0.210	0.152	1.382	0.200
Cars	0.023	0.121	0.190	0.854	0.261	0.075	3.487	0.007**
Service (total)	0.109	0.177	0.613	0.555	−0.037	0.095	−0.384	0.710
Supermarket	−0.073	0.098	−0.737	0.480	0.189	0.092	2.060	0.069
Shopping mall	−0.028	0.086	−0.322	0.755	0.147	0.054	2.723	0.024*
Convenience store	−0.082	0.233	−0.351	0.733	−0.113	0.183	−0.615	0.554
Clothes	0.176	0.142	1.241	0.246	0.307	0.172	1.789	0.107
Food/drink	−0.033	0.365	−0.090	0.930	0.710	0.202	3.516	0.007**
Lifelines	0.009	0.039	0.229	0.824	−0.055	0.040	−1.360	0.207
Supplement	0.276	0.171	1.613	0.141	0.004	0.052	0.071	0.945
Non-prescription drug	−0.015	0.050	−0.296	0.774	0.145	0.063	2.303	0.047*
Medical	0.068	0.162	0.419	0.685	0.055	0.092	0.592	0.568
Pub	−0.014	0.085	−0.166	0.872	−0.085	0.042	−2.005	0.076
Travel agency	−0.102	0.072	−1.416	0.191	0.037	0.032	1.160	0.276
Hotel	0.019	0.047	0.400	0.699	0.028	0.029	0.975	0.355
Cosmetic	−0.066	0.092	−0.724	0.487	−0.049	0.032	−1.546	0.156
Communication	0.136	0.214	0.636	0.541	0.521	0.216	2.413	0.039*
Contents distribution	−0.004	0.073	−0.053	0.959	0.139	0.051	2.717	0.024*
Hausman X^2^=	42.03			*(p < 0*.01)	96.99			*(p < 0*.01)

β, coefficient value; SE, standard error; T, T vales; X^2^, Chi-square value by Hausman's likelihood ratio test.

* p < 0.05,

** p < 0.01.

**Table 4:** Relationship between personal consumption expenditures and SDRs for males and females in their twenties in the non-metropolitan region during the COVID-19 pandemic (between March 2020 and December 2021).

**Factor**	**Males**				**Females**			
	**β**	**SE**	**T**	***p***	**β**	**SE**	**T**	***p***
Credit card	0.051	0.097	0.525	0.603	−0.159	0.065	−2.459	0.019*
Tangibles	0.428	0.259	1.651	0.107	0.009	0.171	0.052	0.959
Retail	−0.019	0.075	−0.249	0.805	−0.007	0.051	−0.136	0.893
Cars	−0.049	0.038	−1.273	0.211	0.008	0.024	0.326	0.746
Service (total)	−0.040	0.030	−1.320	0.195	0.017	0.032	0.513	0.611
Supermarket	−0.146	0.043	−3.384	0.002**	−0.046	0.032	−1.414	0.166
Shopping mall	−0.027	0.026	−1.048	0.302	−0.004	0.016	−0.237	0.814
Convenience store	0.021	0.040	0.521	0.606	0.030	0.044	0.670	0.507
Clothes	−0.020	0.036	−0.572	0.571	0.101	0.036	2.827	0.008**
Food/drink	−0.253	0.102	−2.472	0.018*	0.055	0.069	0.801	0.428
Lifelines	0.033	0.013	2.568	0.015*	−0.020	0.013	−1.568	0.126
Supplement	0.013	0.030	0.431	0.669	0.029	0.027	1.075	0.289
Non-prescription drug	0.018	0.012	1.510	0.140	−0.017	0.018	−0.944	0.351
Medical	0.048	0.016	2.994	0.005**	−0.002	0.017	−0.099	0.922
Pub	−0.058	0.021	−2.765	0.009**	−0.029	0.013	−2.323	0.026*
Travel agency	−0.003	0.002	−1.938	0.061	−0.001	0.002	−0.534	0.596
Hotel	−0.019	0.010	−1.841	0.074	0.020	0.011	1.832	0.075
Cosmetic	0.003	0.010	0.344	0.733	−0.007	0.011	−0.642	0.525
Communication	−0.027	0.089	−0.308	0.760	0.303	0.077	3.917	0.000**
Contents distribution	0.017	0.020	0.848	0.402	0.045	0.015	3.077	0.004*
Hausman X^2^ =	72.70			*(p < 0*.01)	81.43			(*p* < 0.01)

β, coefficient value; SE, standard error; T, T vales; X^2^, Chi-square value by Hausman's likelihood ratio test.

* p < 0.05,

** p < 0.01.

## Discussion

### Features of transformed personal consumption during the COVID-19 pandemic

The present study identified the features of transformed personal consumption behaviors and increased SDRs during the COVID-19 pandemic in Japan. Annual GDP in 2020 and 2021 was 3.7% less and 0.7% more than the values of the previous year; therefore, GDP in Japan during the pandemic was decreased compared to the pre-pandemic period (60). In the present study, the total values of personal consumption expenditures during the pandemic also decreased compared to the pre-pandemic period. However, the decrease of more than 25% compared to the predicted values were expenditures on pubs, travel agencies, and hotels. It is reasonable to understand that these drastically decreasing personal consumptions are the result of governmental restriction measures against expanding the COVID-19 pandemic and/or individual self-suppression. Indeed, these personal expenditures were increased by governmental countermeasures “Go-To-Travel campaigning” (from July/2020 to March/2021) and “Go-To-Eat campaigning” (from September/2020 to November/2020) against deteriorations of the respective tourism and food services industries (61). Therefore, the present study could add a novel candidate for suicide risks compared to the findings of previous studies associated with suicide, since the results in this study suggest that the changing individual consumption behaviors (as a result of a transformed lifestyle) probably contributed to the increasing suicides during the pandemic. Decreasing expenditures on out-of-home recreations, such as travel agencies, hotels and pubs were initiated in January, February, and March 2020, respectively. Therefore, it is possible that extraordinary recreation (travel) was refrained first, followed by suppression of daily recreation (pub) (62). These decreasing out-of-home recreations were replaced by home-based recreation, such as content distribution. Notably, this transformed consumption and recreational behaviors were observed before the “COVID-19 state of emergency” (first governmental stay-at-home order) (April-May/2020). Therefore, transformed individuals' consumption/recreation behavioral lifestyles should be considered as not induced by government measures but by individual factors. Indeed, the nighttime population in Tokyo had already begun decreasing before the governmental stay-at-home order (40).

The transformed lifestyle due to individual factors could also be demonstrated by the changing daily necessities purchase behaviors, which changed during the pandemic, decreasing consumption in shopping malls and convenience stores but increasing in supermarkets. Furthermore, personal consumption of supplements and non-prescription drugs, which are available *via* online shopping, also increased, whereas expenditures for medical expenses that require visits to medical facilities decreased (63, 64). These results suggest that individuals reduced visits to buy daily necessaries and purchased more on each visit for reducing the risks of COVID-19 exposure (41, 42, 44). Individuals also turned to online shopping, resulting in considerable changes to retail and commerce (41, 44, 65).

Regarding the recreational expenditures that have displayed drastic changes, individuals seemed to replace out-of-home recreations with home-based recreations, such as content distribution. Therefore, the shrinking of the living space (extended time at home) progressed with the extension of the pandemic duration. In non-metropolitan regions, communication expense, which was expected to be an alternative to communication with others due to decreasing outing opportunities, increased in 2020, but this effect size was very small. However, unexpectedly, communication expenses in metropolitan regions throughout the pandemic and in the non-metropolitan region in 2021 decreased. These fluctuations regarding communication expenses indicate that the isolation of individuals from the community may have progressed.

### Fixed-effects of personal consumption on SDRs during the COVID-19 pandemic

The present study indicated three features of SDR during the pandemic in Japan.

1. Increasing females SDR was more predominant than that of males.
2. Increasing SDR in the non-metropolitan region was more predominant than that in the metropolitan region.
3. Increasing SDR of the younger population was more predominant than that of the elderly.

The specific feature of SDRs during the pandemic included increased female SDR compared to that of males. The present study demonstrated that generally personal consumption during the pandemic decreased, with some exceptions, compared to those of the pre-pandemic period. Furthermore, the Japanese complete unemployment rate during the pandemic increased (from 2.4% before the pandemic to 2.8% during the pandemic), whereas the increase in Japan was relatively slight compared to those of the Western countries (66, 67). Japan has experienced three major economic crises in the last 30 years. Following the collapse of the asset bubble in 1991 and immediately following the 1997 Asian economic crisis, suicide mortality in Japan drastically increased in 1998, but the increase in male suicide mortality was predominantly rather than that of females (68, 69). Contrary, the decreasing trends of suicide mortality were not affected by the financial crisis of 2008 (32, 33, 57). These three economic crises deteriorated the socioeconomic status and employment rate in Japan. Therefore, the predominantly increasing SDR of females compared to that of males during the pandemic is peculiar in light of the previous experiences associated with the economic crisis in Japan. Based on these, in order to identify the mechanisms of increasing SDR during the pandemic, the present study statistically analyzed the fixed-effect of personal consumption expenditures (not only as a socioeconomic indicator but also as an individual lifestyle indicator) on SDR using fixed-effect of a hierarchical linear regression model with robust standard error.

The expenditures on content distribution increased exceptionally in the personal consumption during the pandemic. In the present study, fixed-effects of expenditures on content distribution were positive impacts on females' SDR but did not impactful on males' SDR. Various findings have already been reported to support that the increasing expenditures on content distribution contributed to increasing female SDR compared to male SDR during the pandemic. Online interactions have played an increasingly larger role in social situations during the pandemic (70); however, the form of the internet exhibits a difference in terms of whether it relates to mental health for males and females (71, 72). Especially, passive internet use is related to increased depressive symptoms in females predominantly (71, 72). Therefore, indirectly, these results possibly support the possibility that increased expenditures on content distribution contributed to selectively increased female SDR.

The factors behind the increasing SDR in the non-metropolitan region over that in the metropolitan region were also detected. It has been established that deviating alcohol consumption contributes to a major risk factor for death/disease and suicide (73). Especially, during the pandemic, alcohol use has been also considered a biologically and socio-psychologically deteriorating prognostic factor for COVID-19 (74, 75). Alcohol consumption tended to elicit behavior that promotes lower compliance to COVID-19 preventive measures (74). Indeed, individual mobilizations with expenditures to pubs across the prefectural borders played important roles in expanding the COVID-19 pandemic in Japan (76). Furthermore, a recent scoping review study revealed that rural were associated with an increased likelihood of hazardous alcohol use or alcohol-related harm compared to urban (73). In contrast to previous findings regarding alcohol consumption behaviors, the present study demonstrated that decreasing expenditures on pubs, which drastically decreased during the pandemic, impacts increasing SDRs in the non-metropolitan region but is not impactful SDR in the metropolitan region. The contradiction between the present demonstration and previous findings associated with alcohol consumption behaviors suggests that pubs in the non-metropolitan in Japan might have functioned as an important social gathering and/or communication place with other persons, rather than merely serving alcohol.

About 30% of young individuals realized the transformation of their daily routine behaviors due to worrying about someone, who was infected with COVID-19 and a reduction of times contacting with other persons during the pandemic, leading to the deterioration of their mental health (71). Therefore, we speculated that internet/mobile communications played an important role as a tool to compensate for the diminished contact with others individuals during the pandemic. However, contrary to our expectations, increasing expenditures on communication expenses is positively related to increasing SDRs of males and females in both metropolitan and non-metropolitan regions. Especially, the SDRs of 20s females in both regions positively related to increasing communication expense but those of 20s males did not relate. The positive impact of communication expense on suicide can be explained by the fact that WHO formally recognized addiction to digital technology (connected devices) as a worldwide problem, where excessive online activity and internet use lead to various disturbances of mental health (77). Furthermore, the expenditure on communication decreased in metropolitan during the pandemic, whereas that in the non-metropolitan region weakly increased in 2020, but converted to a weak decrease in 2021 (the reduction was predominant in the metropolitan region compared to the non-metropolitan region). Fortunately, in the metropolitan region, decreasing the expenditures on communication expenses probably mitigated its positive impacts on SDRs, but in the non-metropolitan region, the positive impacts on SDRs were relatively lesser mitigated due to the necessities of internet/mobile communication for contact with other persons. In other words, internet/mobile communication alone cannot compensate/mitigate isolation induced by the reduction of the opportunities for direct contact with other persons.

Although the present study could not detect the crucial factors that contributed to increased female SDRs during the pandemic, especially SDRs of 20s females in non-metropolitan regions, the combinations of targets among the fixed-effects of expenditures on content distribution, pubs, and communication expense elicits the targets of increasing SDRs of 20s females in the non-metropolitan region. The development of suicide risk is composed of various complicated factors, such as socioeconomic, socio-psychological, clinical, and biological factors (78). Based on the involvement of complex multiple risk factors for suicide, the results in this study are rational. Individuals in their 20s experience drastic changes in their living environment/place in metropolitan regions due to continuation to university/graduate school and employment (79, 80). Individuals, who have undergone changes in their living environments/places due to higher education and new employments, usually make efforts to explore and adapt to their new communities; however, these efforts cannot be fully performed due to telecommunication, online lessons, and restrictions of pubs. Indeed, the mental health condition of first-year university students in Japan deteriorated and the number of high-risk students with suicidal ideation increased during the pandemic (81). A working population survey study in Japan reported that the risk factors for increasing suicidal ideation during the pandemic were being a female, aged below 30 years old, high education, and having no pre-existing mental health conditions (82). Considering these previous findings, internet/mobile communications alone, as alternative communication tools, cannot fully improve the suffering isolation/loneliness situations of individuals facing a turning point in their lives (such as individuals who have been forced to change to a new society/community due to higher education and/or employment). Therefore, the Internet-based “new normal” lifestyle (39), which is attracting attention as a new lifestyle during post-pandemic, might generate a new high-risk group for suicide, freshmen/freshwomen who are facing a turning point in their lives.

Traditionally, the SDRs of elderly males (50s−80s), which have been the predominant risk for suicide in Japan, decreased compared to the predicted SDR. Particularly, male SDR in 2021 was lower than in 2020. The Tokyo Olympics, which was scheduled to be held in 2020, was postponed to be held in 2021, whereas it was largely held behind closed doors with no public spectators permitted due to the declaration of a “State of emergency” in the Kanto metropolitan region for response to the pandemic. In fact, no significant changes in personal consumption were observed before and after the Olympics. Therefore, it cannot be denied that the hosting of the Olympics contributed to decreasing male SDR in 2021 *via* positive socio-psychological impacts, it is difficult to speculate on positive socioeconomic impacts. It has been well known that the “Regional comprehensive suicide prevention program” and “Emergency Fund to Enhance Community-Based Suicide Countermeasures” have contributed to the decreasing suicide mortality in Japan (32, 33, 57, 69, 83). MHLW quickly budgeted for “Suicide prevention measures in response to the COVID-19 pandemic,” in both 2020 and 2021, since MHLW speculated the increasing suicides due to the socio-psychological/socioeconomic deteriorations induced by the COVID-19 pandemic (21). The “Suicide prevention measures in response to the COVID-19 pandemic” was the enhancement of regional suicide prevention programs generated by the public service corporations, social welfare corporations, and non-profit organizations that have contributed to regional suicide prevention programs before the COVID-19. Taken together with the previous findings that both “Regional comprehensive suicide prevention program” and “Emergency Fund to Enhance Community-Based Suicide Countermeasures” has contributed to the decreasing suicide mortality of elderly males (33), “Suicide prevention measures in response to the COVID-19 pandemic” was probably involved in the decreasing SDR of elderly males during the pandemic.

The expenditure to travel agency, which also drastically decreased during the pandemic, was not related to male SDRs in both regions or female SDR in the non-metropolitan region, whereas the increasing expenditures to travel agency was positively related to female SDR in the metropolitan region. The positive impact of increasing expenditure to travel agencies on female SDR in metropolitan regions is probably due to the synchronization with the transient increasing SDRs observed in October/2020 of wide age ranges in the metropolitan region. During October/2020, the transiently/drastically increasing female SDR is considered to be composed of increasing copycat suicide by hanging at their home (12–15). The increasing hanging suicide at home is one of the peculiar features of the increase in suicides in Japan during the pandemic (15). In contrast, the selection of a place away from the daily living environment is well known to be a major behavior for suicide victims. Indeed, intentional fatalities in the form of suicide accounted for the second highest (about 20%) number of all fatalities in USA National Parks (84, 85). Further discussion regarding the relationship between suicides and travel is beyond the scope of the present study since we could not get any available data on the number of suicides during or after travel, whereas the relationship between travel and suicide possibly provides important information for the reconstruction of the tourism industry after the pandemic.

### Strengths and limitations

The present study had several strengths and limitations. The most important strength is that the study design allowed us to identify the relationship between personal consumption behaviors disaggregated by prefecture with monthly suicide mortalities disaggregated by prefecture, sex, and age during the COVID-19 pandemic, using fixed-effect of hierarchical linear regression models with robust standard error. However, there are some limitations to this study. JCB Consumption NOW database publishes the monthly expenditures of personal consumption disaggregated by prefectures but does not publish the values disaggregated by sex or age. Therefore, a further study that analyses the relationship between SDR and personal consumption expenditure disaggregated by sex and age is needed to clarify the relationship between personal consumption behaviors and suicide mortality.

## Conclusions

The present study identified the fluctuation patterns in personal consumption expenditures and suicide mortality during the COVID-19 pandemic in Japan. Similar to other Western countries, the Japanese government has quickly responded to pandemic-related socio-psychological deterioration *via* “Suicide prevention measures in response to the COVID-19 pandemic,” which were developed in accordance with the “Regional comprehensive suicide prevention program” and “Emergency Fund to Enhance Community-Based Suicide Countermeasures.” “Suicide prevention measures in response to the COVID-19 pandemic” did not appear to decrease the female SDR or the SDR of the younger population, which were not major targets of governmental suicide prevention programs for the past decade. Ironically, it appears that the measures were associated with decreasing the SDR of elderly males, which has been a major target of governmental suicide prevention programs. Therefore, in order to respond to the newly increasing SDR among females and younger populations during the pandemic, it is necessary to rapidly improve the “Suicide prevention measures in response to the COVID-19 pandemic.” This improvement should be also effective for the prevention of suicide in the “new normal,” a concept that has attracted attention during and after the pandemic.

## Data availability statement

The original contributions presented in the study are included in the article/Supplementary material, further inquiries can be directed to the corresponding author/s.

## Ethics statement

Ethical approval and informed consent were exempted by the Medical Ethics Review Committee of Mie University because the present study only used publicly available data.

## Author contributions

MO conceptualized the study, contributed to the study design and methodology, drafted, and reviewed the manuscript. YK and RM contributed to the study design and methodology, verified the underlying data, performed the statistical analysis, generated figures, and drafted and reviewed the manuscript. EM contributed to the study design and methodology and coordinated extraction of the data. TS conducted the literature review, contributed to the methodology, and drafted and reviewed the manuscript. All authors contributed to the article and approved the submitted version.

## Funding

This study was supported by Regional Suicide Countermeasures Emergency Enhancement Fund of Mie Prefecture (2021-40).

## Conflict of interest

The authors declare that the research was conducted in the absence of any commercial or financial relationships that could be construed as a potential conflict of interest.

## Publisher's note

All claims expressed in this article are solely those of the authors and do not necessarily represent those of their affiliated organizations, or those of the publisher, the editors and the reviewers. Any product that may be evaluated in this article, or claim that may be made by its manufacturer, is not guaranteed or endorsed by the publisher.
