# Supplementary material for: Analyzing the changing relationship between personal consumption and suicide mortality during COVID-19 pandemic in Japan, using governmental and personal consumption transaction databases
Source: Front Public Health. 2022 Sep 7;10:982341. doi: 10.3389/fpubh.2022.982341 (PMC9489934; doi:10.3389/fpubh.2022.982341)
Supplement: Supplementary file 1 [file Data_Sheet_1.docx]

Supplementary Material

**Supplementary Figure 1.** Analysis of joinpoint of annual SDR during pre-pandemic period (between 2009 and 2019) (A) and observed and predicted SDRs using seasonal multiplicative regression model derived from January/2017 to December/2019 (B) in Japan. Panel A: The annual percent change (APC) values in SDR were -5.53 (red line: 2009-2017) and -2.42 (blue line: 2017-2019) (p<0.05). Ordinates indicate the standardized suicide mortality (per 100,000 people), and abscissas indicate the year. Panel B: Black lines indicate observed SDRs. Blue, green, and red lines indicated expected by “trend variation*cyclical variation”, “trend variation*cyclical variation*irregular variation” and “trend variation*cyclical variation* seasonal variation*irregular variation” using seasonal multiplicative regression model. The correlation between observed and predicted SDRs were significant (p<0.001). Ordinates indicate the standardized suicide mortality (per 100,000 people), and abscissas indicate the month.


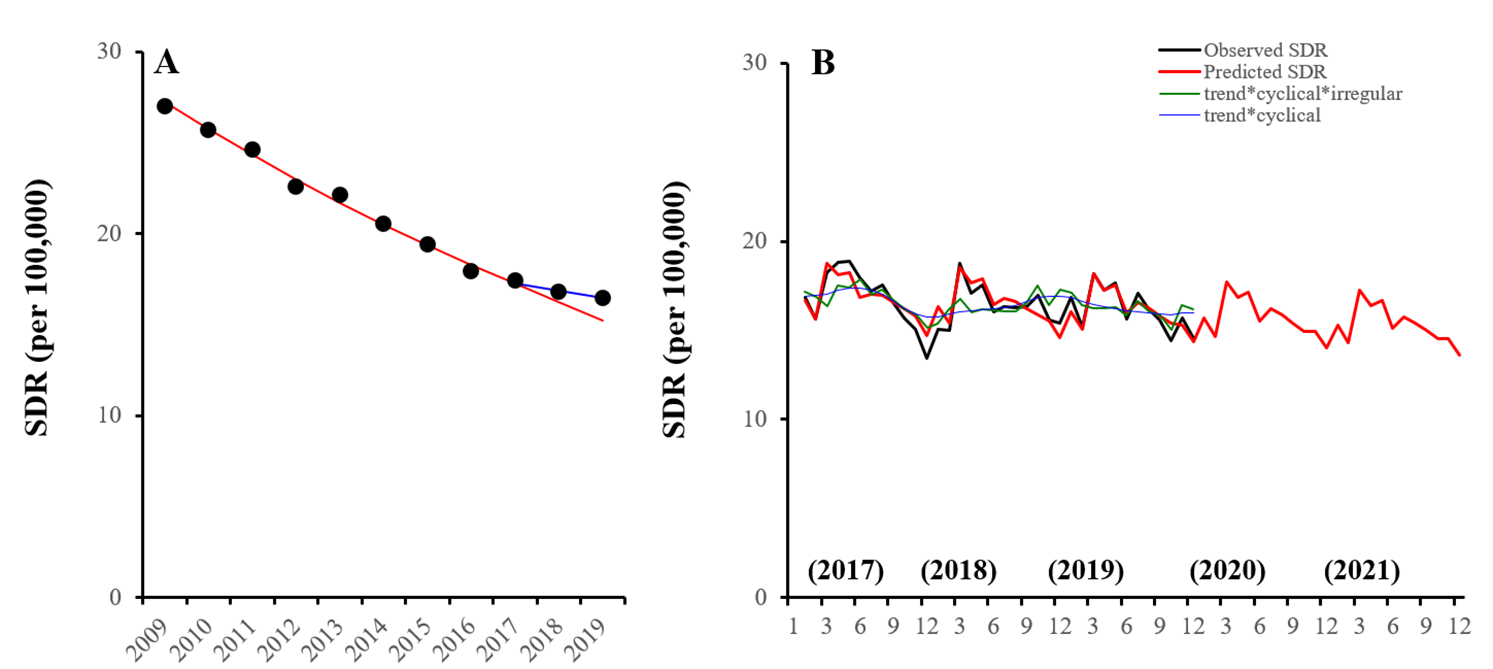


**Supplementary Figure 2.** Average of annual SDR disaggregated by gender, males (A1-H1) and females (A2-H2), region, all 47 prefectures (All), metropolitan (Metro) and non-metropolitan (Non) regions, and ages, 10s (A1-A2), 20s (B1-B2), 30s (C1-C2), 40s (D1-D2), 50s (E1-E2), 60s (F1-F2), 70s (G1-G2) and 80s (H1-H2), during the COVID-19 pandemic (2020–2021). Red and blue bars indicate the average of relative observed SDR per predicted SDR in 2020 and 2021, respectively. Ordinates indicate the mean±95%CI of relative annual SDRs (%). *p<0.05, **p<0.01; relative to the predicted SDR, @p<0.05, @@p<0.01; relative to 2020, and #p<0.05, ##p<0.01; relative to metropolitan region using a linear mixed-effects model with Scheffe’s post-hoc test.


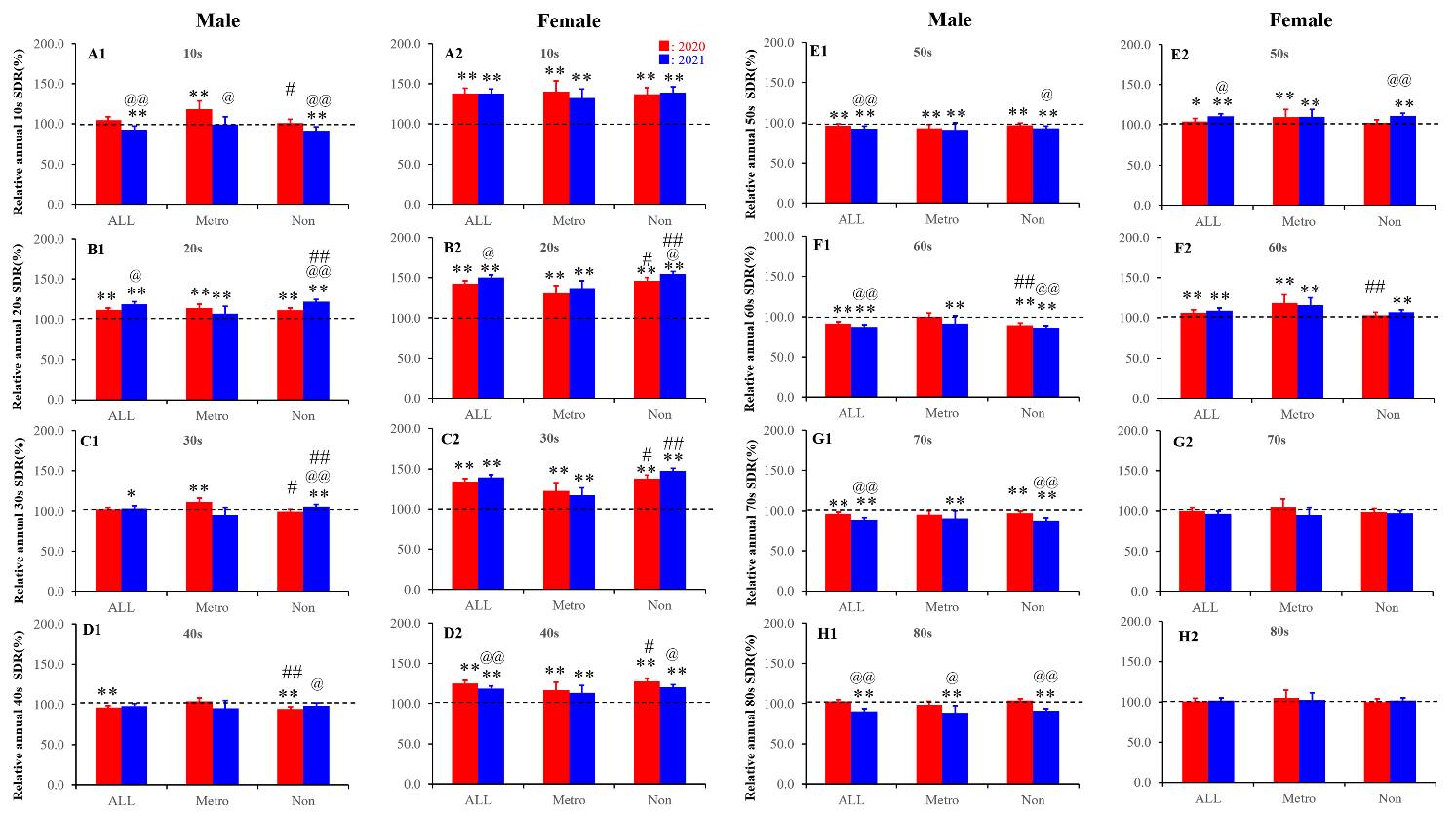


**Supplementary Figure 3.** Fluctuations of monthly expenditures during the COVID-19 pandemic in Japan. Ordinates indicate the relative personal expenditures between January/2020 and December/2021 per that in March 2016 (%), and abscissas indicate the month. Dotted, blue and red lines indicate the average of predicted and observed personal expenditures in 2020 and 2021, respectively. Brown areas, blue and red bars indicate the 95%CI of predicted and observed personal expenditures in 2020 and 2021, respectively. *p<0.05, significant change of monthly suicide mortality compared to predicted vales using a linear mixed-effects model with Scheffe’s post-hoc test. ↑and↓indicate significantly higher and lower observed annual SDR compared to predicted annual SDR, respectively.


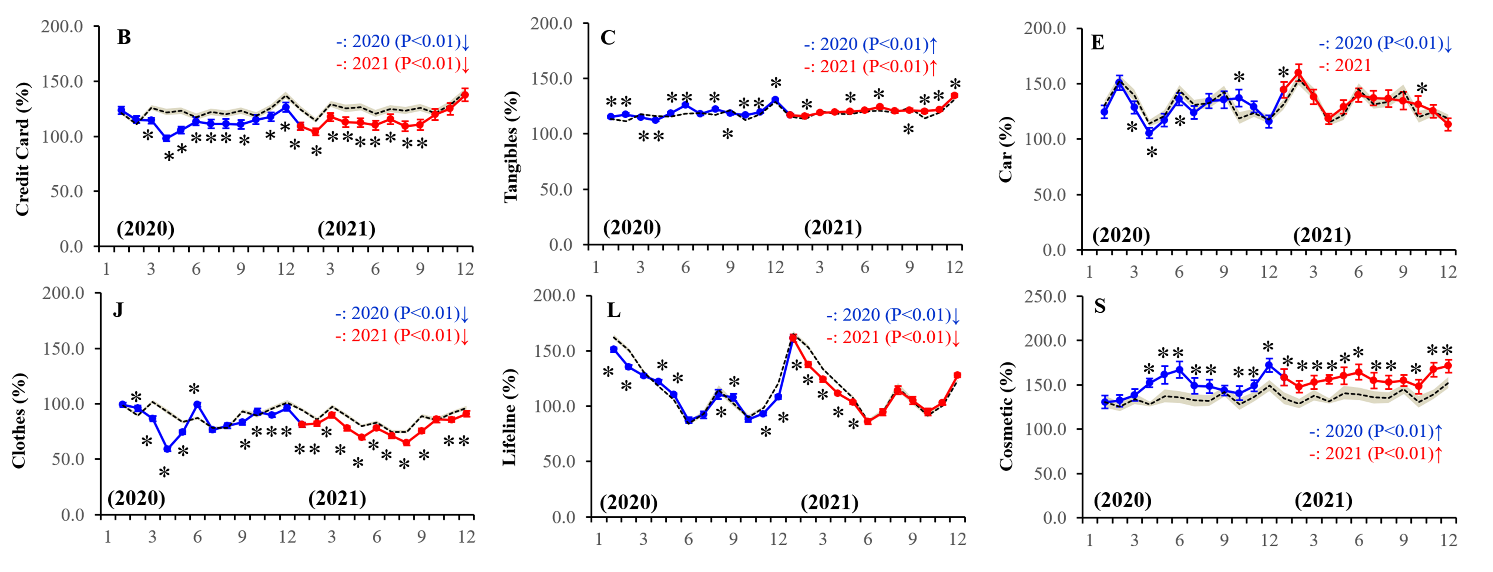


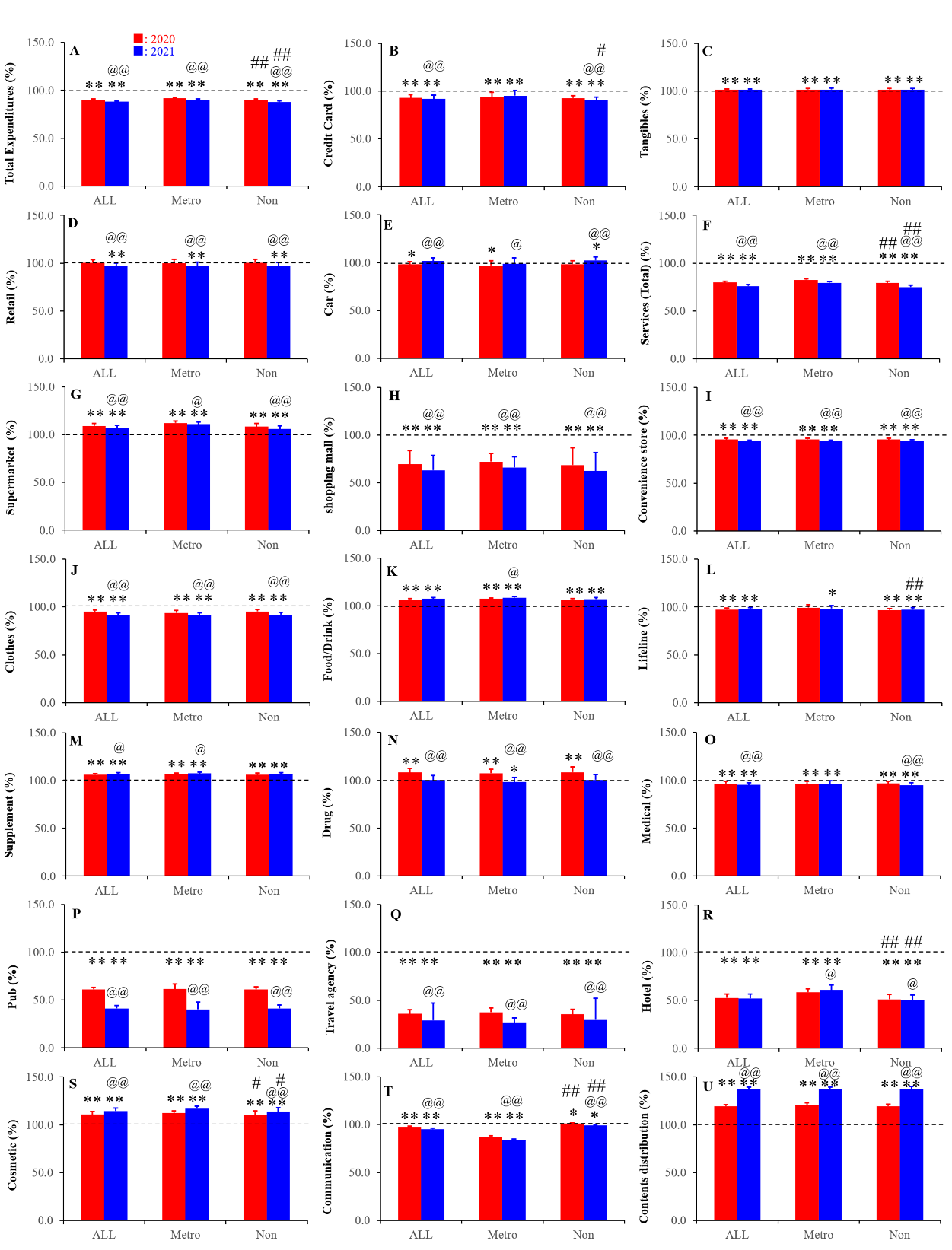
**Supplementary Figure 4.** Average of personal consumption expenditures disaggregated by regions, all 47 prefectures (All), metropolitan (Metro) and non-metropolitan (Non) regions, during the COVID-19 pandemic (2020–2021). Red and blue bars indicate the average of relative personal expenditures per predicted values in 2020 and 2021, respectively. Ordinates indicate the mean±95%CI of relative personal expenditures (%). *p<0.05, **p<0.01; relative to the predicted value, @p<0.05, @@p<0.01; relative to 2020, and #p<0.05, ##p<0.01; relative to metropolitan region using a linear mixed-effects model with Scheffe’s post-hoc test.
